# Cognitive Tagging of Neurons: CREMediated Genetic Labeling and Characterization of the Cells Involved in Learning and Memory

**Published:** 2018

**Authors:** O. I. Ivashkina, N. S. Vorobyeva, A. M. Gruzdeva, M. A. Roshchina, K. A. Toropova, K. V. Anokhin

**Affiliations:** Department of Neuroscience, National Research Center “Kurchatov Institute”, Akademika Kurchatova pl. 1, Moscow, 123182, Russia; Center for Neural and Cognitive Sciences, Lomonosov Moscow State University, Leninskie Gory 1 , bldg. 27, Moscow, 119991, Russia; Institute of Higher Nervous Activity and Neurophysiology of RAS, Butlerova Str. 5A, Moscow, 117485 , Russia; Laboratory for Neurobiology of Memory, P.K. Anokhin Institute of Normal Physiology, Baltiyskaya Str. 8, Moscow, 125315, Russia

**Keywords:** brain, neuronal networks, learning, immediate early genes, Cre-recombination, genetic labeling of neurons

## Abstract

In this study, we describe use of Cre-mediated recombination to obtain a permanent genetic labeling of the brain neuronal networks activated during a new experience in animals. This method utilizes bitransgenic Fos-Cre-eGFP mice in which a green fluorescent protein is expressed upon tamoxifen-induced Cre-recombination only in the cells where immediate early gene *c-fos *expression takes place due to the new experience. We used the classical fear conditioning model to show that *ex vivo *microscopy of the eGFP protein in Fos-Cre-eGFP mice enables mapping of the neurons of the various brain regions that undergo Cre-recombination during acquisition of a new experience. We exposed the animals to the new environment in brief sessions and demonstrated that double immunohistochemical staining enables a characterization of the types of neocortical and hippocampal neurons that undergo experience-dependent Cre-recombination. Notably, Fos-Cre-eGFP labeled cells appeared to belong to excitatory pyramidal neurons rather than to various types of inhibitory neurons. We also showed that a combination of genetic Cre-eGFP labeling with immunohistochemical staining of the endogenous c-Fos protein allows one to identify and compare the neuronal populations that are activated during two different episodes of new experiences in the same animal. This new approach can be used in a wide spectrum of tasks that require imaging and a comparative analysis of cognitive neuronal networks.

## INTRODUCTION

The study of the mechanisms of formation and functioning of the neural networks involved in cognitive functions is one of the most important aspects of modern neuroscience. The development of new experimental methods for cell-resolution visualization of the neural substrates of cognitive processes throughout the whole brain plays a key role in addressing these problems.

Detection of immediate early gene expression is a classical method used to identify the neuronal populations involved in various types of cognitive activity in animals [1-3]. IEG is a family of genes that are rapidly activated in the cell through intracellular signaling cascades in response to certain external influences. This family includes genes that encode transcription factors, structural synaptic proteins, cytoplasmic enzymes, etc. [4]. Expression of some IEGs, for example *c-fos*, *zif268 *and *arc*, is induced in animals through a new experience and plays a key role in plastic rearrangements and the formation of long-term memory, storing the individual experience [1, 3, 5, 6]. These properties of IEGs form the basis for the methods of immunohistochemical and *in situ *hybridization mapping of the brain, which make it possible to identify the cognitive neural networks activated during learning [2, 7-9].

However, identification of IEG products using immunohistochemistry or *in situ *hybridization is laborious and limited to a narrow time window after cognitive exposure. Visualization of the populations of cognitively active neurons using expression of fluorescent protein genes controlled by IEG promoters is a promising way to address some of these problems. The use of controlled transgenic systems makes it possible to obtain mice in which the fluorescent protein is synthesized only in those neurons where IEG, for example *c-fos*, was expressed at a certain time [10]. In recent years, these technologies have become increasingly widespread. However, they suffer from a significant limitation: labeling of the population of cognitively active neurons is limited by the lifetime of the fluorescent protein.

Permanent genetic labelling of the population of neurons involved in a certain cognitive task may be a solution to this problem. The system of site-specific recombinases widely used in neurobiology [11-13], in particular the one with modified Cre-recombinase of P1 bacteriophage, which is bound to the mutant ligand-binding domain of the estrogen receptor, can be used for this purpose [14, 15]. Cre-recombinase recognizes homotypic loxP sites and excises the DNA flanked by these sites in the presence of tamoxifen, a synthetic estrogen antagonist. LoxP sites usually flank the STOP codon located before the reporter gene sequence that encodes the fluorescent protein (GFP, tdTomato, etc.), β-galactosidase, or channelrhodopsin [13, 16]. At present, dozens of transgenic mouse strains have been developed where Cre-recombinase is selectively expressed in different tissues or different cell types; for example, in certain types of interneurons [17] or at certain stages of neuronal progenitor development [13]. Thus, the technology of genetic labelling of neurons with Cre-recombinase is currently well developed, but it is primarily used for anatomical mapping of the nervous system or for embryonic research.

The work of Guenthner et al. [18], who developed transgenic mice lines expressing Cre-recombinase under the control of the IEG promoters *c-fos *and *arc, *was a methodological innovation. Cross-breeding of Fos-Cre or Arc-Cre mice with reporter mice carrying the red fluorescent protein tdTomato for the first time provided permanent genetic labeling of neurons with activated IEGs [18].

We generated double transgenic Fos-Cre-eGFP mice which were used in this work to investigate the morphological and molecular phenotype of the neurons of various brain structures undergoing Cre-recombination when animals acquire a new experience. We also tested for the first time the possibility of using Fos- Cre-eGFP transgenic mice to label and compare two populations of neurons activated in the same animal in two different situations when gaining new experience.

## EXPERIMENTAL

**Preparation of bitransgenic Fos-Cre-eGFP mice**

B6.129(Cg)-Fostm1.1(cre/ERT2)Luo/J transgenic mice (The Jackson Laboratory, stock Number: 021882) bearing Cre-recombinase under the *c-fos *promoter were interbred with TG(CAG-Bgeo/GFP)21Lbe/J mice (The Jackson Laboratory, Stock No. 003920) carrying the enhanced green fluorescent protein gene eGFP under loxP sites in order to produce Fos-Cre-eGFP mice. The mice were managed in individually ventilated cells, five animals per cell, with water and food ad libitum and a light cycle of 12/12 h. All experiments were carried out in accordance with the requirements of Order No. 267 of the Ministry of Health of the Russian Federation (19.06.2003) and also with the directive of the local ethical Committee on Biomedical Research of the Research Center of the Kurchatov Institute (Protocol No. 1 of 09/07/2015).

**Genotyping**

The transgene was in a heterozygous state in both parental lines, and, therefore, genotyping for each transgene was used to identify bitransgenic animals. Tissue samples were collected from mice at the age of two months, and DNA was isolated by lysing with a proteinase K solution (Sigma) in 1% SDS buffer (Helicon), followed by precipitation with 96% ethanol. The DNA precipitate was dissolved in a TE buffer. The resulting DNA was used for a polymerase chain reaction (PCR) with primers to Cre-recombinase and eGFP in a ScreenMix medium (ZAO Eurogen). The following primers were used: for Cre – CACCAGTGTCTACCCCTGGA (common forward sequence for the wild type and transgene), CGGCTACACAAAGCCAAACT (reverse wild-type sequence), CGCGCCTGAAGATATAGAAGA (reverse transgene sequence); for GFP – TGGACGGCGACGTAAACGGC (first transgene sequence), GGCGGTCACGAACTCCAGCA (second transgene sequence), CTAGGCCACAGAATTGAAAGATCT (forward sequence of the internal positive control), GTAGGTGGAAATTCTAGCATCATCC (reverse sequence of the internal positive control). PCR conditions are shown in *table*. PCR products were detected in 2.5% agarose gel in a TAE buffer with ethidium bromide.

**Table:** PCR conditions for genotyping

Step	Temperature, °C	Time	Remark
1	94	2 min	
2	94	20 s	
3	65	15 s	Decrease in temperature by 0.5°C per cycle
4	68	10 s	
5			Repeating steps 2 to 4 for 10 cycles
6	94	15 s	
7	60	15 s	
8	72	10 s	
9			Repeating steps 6 to 8 for 28 cycles
10	72	2 min	
11	10		Stop

**Induction of Cre-recombination with tamoxifen**

A single intraperitoneal injection of tamoxifen (Sigma) dissolved in corn oil (Sigma) was used at a dose of 150 mg/kg to induce Cre-recombination as previously described [18]. For experience-induced Cre-recombination, tamoxifen was injected 24 hours before the animal gained a new experience.

**Context exploration**

For new context exploration, the mice were placed in an experimental chamber for 5 minutes and allowed to freely explore it. It is known that such an exploration leads to the formation of long-term memory in animals [19].

Two contexts were used in this work. Context A was a chamber illuminated with diffused white light (average illumination level 87 lux) size 20 × 30 × 20 cm and equipped with an electrode floor; the noise level in the chamber was 7 dB. The chamber was wiped with a 40% solution of ethyl alcohol before placing each animal in it. Context B was a chamber size 20 × 15 × 20 cm with an electrode floor; the camera was illuminated only with a near-infrared light source, which is invisible to mice; the noise level in the chamber was 25 dB. The chamber was wiped with a 3% acetic acid solution before placing each animal in it.

**Immediate footshock**

Footshock (FS) was applied 3 days after the exploration of context A. For this purpose, the mice were placed in context A or context B and immediately administered FS with an intensity of 1 mA and duration of 2 seconds and then immediately returned to their home cage.

**Fear conditioning (FC) training**

For the purpose of FC training, the mice were placed in context A, followed by a sound signal (90 dB, 5 kHz) lasting 30 s for 120 seconds. The last 2 seconds of sound were combined with a 1 mA FS. The mice were returned to their home cage 30 seconds after the end of the FC.

**Experimental groups**

Two groups of animals were used to evaluate the baseline and experience-induced eGFP expression: “Learning” group animals (*n *= 4) were fear-conditioned as described above; “control” group mice (*n *= 4) stayed in their home cages and were not exposed to any influences.

The animals that explored context A for 5 min (*n *= 5) were taken to determine the phenotype of the cells undergoing experience-dependent Cre-recombination.

The A – A + FS (*n *= 5) and A – B + FS (*n *= 5) groups were used to identify two populations of cognitively active neurons in one brain. A – A + FS group mice explored context A and then received immediate FS for 3 days in the same context; A – B + FS group mice explored context B and then received immediate FS for 3 days in context B.

**Brain sample harvesting and preparation of floating sections**

Mouse brain samples were obtained 3 days after the animals had been exposed to a new experience which was accompanied by Cre-recombination. In the experiments aimed at identifying two populations of active neurons, mouse brain samples were collected 90 minutes after the second cognitive episode (FS application).

Mice were anesthetized by intraperitoneal injection of 15% chloral hydrate in saline, followed by intracardial perfusion with phosphate buffer and a 4% paraformaldehyde solution in phosphate buffer and brain harvesting. The resulting brain samples were post-fixed with a 4% paraformaldehyde solution in phosphate buffer: 2 h at room temperature and 14–18 h at +4°C. After that, the samples were placed in a phosphate buffer for 2 hours and 50-μm thick frontal sections of the brain were prepared using a Leica VT1200S (Leica) vibratome. Sections were taken at a distance of +2.46 and -1.34 mm from the bregma. The coordinates of the sections were determined using a stereotactic mouse brain atlas [20].

**Detection of the fluorescent protein eGFP and double immunohistochemical staining**

The cells subjected to Cre-recombination in the experiments aimed at determining the baseline and experience-induced eGFP expression were detected based on intrinsic fluorescence of the eGFP protein. The prepared brain sections were placed under cover glass using the Fluoromount™ Aqueous Mounting Medium (Sigma-Aldrich) and digitized as described below.

In the experiments aimed at determining the phenotype and proportion of cells undergoing experience-induced Cre-recombination, as well as the identification of two populations of cognitively active neurons, Cre-recombined cells were detected using immunohistochemistry based on eGFP protein staining. Brain sections were subjected to the permeabilization procedure in a 1% Triton-X100 solution (Sigma) in the phosphate buffer with 5% normal donkey serum (Sigma) and 5% normal goat serum (Abcam) for 60 min, followed by triple washing with a 0.2% Triton-X100 solution in phosphate buffer for 5 minutes and incubation with primary antibodies for 18 hours at +4°C. The following primary antibodies were used in different reactions to identify various markers: eGFP (Rabbit Anti-GFP Antibody, Life Technologies, 1:250 dilution), eGFP (Chicken Anti-GFP Antibody, Aves Labs, 1:500 dilution), c-Fos Goat Anti-c-Fos Antibody (Santa Cruz Biotechnology, 1:150 dilution), GFAP (Chicken Anti-GFAP Antibody, Abcam, 1:500 dilution), NeuN (Mouse Anti-NeuN Antibody, EMD Millipore, 1:500 dilution), CaMKII (Mouse Anti-CaMKII Antibody, EMD Millipore, 1:200 dilution), SOM (Rabbit Anti-Somatostatin Antibody, Santa Cruz Biotechnology, 1:1000 dilution), NPY (Rabbit Anti-Neuropeptide Y Antibody, Novus Biologicals; 1:10000 dilution), and PV (Rabbit Anti-Parvalbumin Antibody, Abcam; 1:10000 dilution). At the end of the incubation, the sections were washed with a 0.2% Triton-X100 solution in phosphate buffer 3 times for 5 minutes. The sections were then incubated with secondary antibodies for 2 hours at room temperature in the dark. The following secondary antibodies were used in different reactions: AlexaFluor® 488 Donkey Anti-Goat Antibody (Life Technologies, 1:500 dilution), AlexaFluor® 488 Goat Anti-Chicken Antibody (Life Technologies, 1:500 dilution), AlexaFluor® 568 Donkey Anti-Rabbit Antibody (Life Technologies, 1:500 dilution), AlexaFluor® 568 Goat Anti-Chicken Antibody (Life Technologies, 1:500 dilution), or AlexaFluor® 568 Donkey Anti-Mouse Antibody (Life Technologies, 1:500 dilution). After incubation with secondary antibodies, sections were washed 3 times for 5 minutes in a 0.2% Triton-X100 solution in phosphate buffer. After the end of staining, the sections were placed under the cover glass using the Fluoromount™ Aqueous Mounting Medium (Sigma-Aldrich). The sections were digitized on a confocal microscope Olympus FV1000BW61WI (Olympus) with ×20 magnification. Each brain section was used to produce 10–13 optical sections with 5 μm increments along the Z axis.

**Counting of eGFP-positive cells and colocalization analysis**

The resulting images were processed, and further analysis was carried out using the Imaris 7.1.0 software (Bitplane). Since eGFP staining is cytoplasmic and involves not only the bodies but also the processes of the cells, the automated counting of eGFP-positive neurons is complicated. Because of this, eGFP-positive cells were marked manually and then counted automatically. NeuN and c-Fos staining is nuclear, and, therefore, automated marking and counting of NeuN-positive and c-Fos-positive cells was used based on the size and threshold level of red-channel color intensity. In the case of eGFP, NeuN, and c-Fos staining, all positive cells whose soma was fully located within the slice along the Z axis were counted; the cells whose soma was located on the upper and lower boundaries of brain sections along the Z axis were not counted. Expert evaluation of eGFP colocalization with NeuN or c-Fos was carried out in three projections for each cell. The cells whose NeuN- or c-Fos-positive nucleus was completely surrounded with eGFP-stained cytoplasm were considered as double positive. Double-positive cells were marked manually and then counted automatically. Positive cells were counted over the entire area of the frontal associative cortex on the brain slice and, in the case of concomitant staining with NeuN, also separately in the layers 1, 2/3, 5, and 6. The boundaries of the frontal association cortex were determined using a stereotaxic mouse brain atlas [20]. The boundaries of the layers were determined according to the Allen Mouse Brain Atlas, http://mouse.brain-map.org/static/atlas. Three sections of the frontal association cortex per animal were analyzed. The results of positive cell counting were averaged for the right and left hemispheres in one section and three sections of one brain.

**Statistical data analysis**

Statistical data processing was carried out with the Prism 7 (GraphPad) software package using a two-sample *t-*test, as well as two-way ANOVA variance analysis or ANOVA variance analysis with repeated measurements and the Sidak *t-*test. The significance level was *p* < 0.05.

## RESULTS AND DISCUSSION

**Baseline and experience-induced expression of the green fluorescent protein in the brain of Fos-Cre-eGFP bitransgenic mice**

**Fig. 1:**
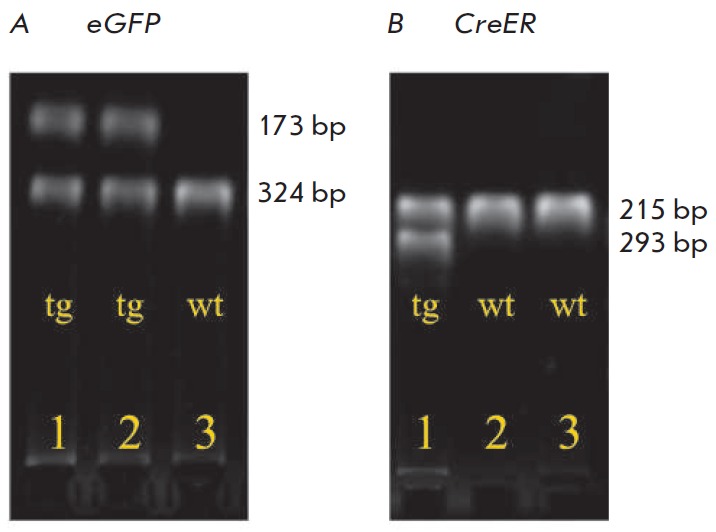
Electrophoretic analysis of PCR products for identification of bitransgenic Fos-Cre-eGFP mice carrying both the eGFP (A) and Cre-recombinase (B) genes. 173 bp – the fragment corresponding to the eGFP transgene; 324 bp – internal positive control; 215 bp – the fragment corresponding to the wild-type Cre-recombinase gene; 293 bp – the fragment corresponding to epy Cre-recombinase transgene; wt – wild-type animal, tg – transgenic animal; 1, 2, 3 – animal number. Only mouse No 1 is bitransgenic.

**Fig. 2:**
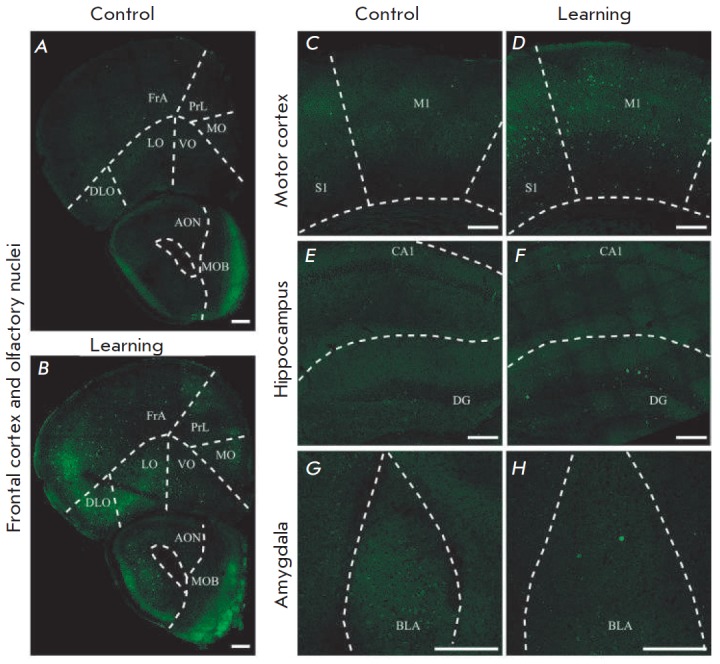
Baseline level of Cre-recombination in the “Control” group naïve mice (A, C, E, G) vs experience-induced Cre-recombination in the “Learning” group of fear-conditioned mice (B, D, F, H). AON – anterior olfactory nucleus; BLA – basolateral amygdala; CA1 – CA1 area of the hippocampus; DG – dentate gyrus of the hippocampus; DLO – dorsolateral orbital cortex; FrA – frontal association cortex; LO – lateral orbital cortex; M1 – primary motor cortex; MO – medial orbital cortex; MOB – main olfactory bulb; PrL – prelimbic cortex; S1 – primary somatosensory cortex; VO – ventral orbital cortex. Scale – 100 μm

Cross-breeding of B6.129(Cg)-Fostm1.1(cre/ERT2) Luo/J and Tg(CAG-Bgeo/GFP)21Lbe/J mice resulted in offspring which partially consisted of bitransgenic animals simultaneously carrying the Cre-recombinase gene under the *c-fos *promoter and an enhanced fluorescent protein eGFP gene under the loxP-flanked stop signal (*Fig. 1*). Transgenic mice were injected with tamoxifen, followed by FC training 24 hours after injection (“Learning” group) or left in the home cells (“Control” group) in order to assess the baseline level of Cre-recombination and experience-dependent induction of recombination. In 3 days, eGFP was detected on the mouse brain sections (*Fig. 2*). The neurons where the green fluorescent protein was detected were the neurons involved in the activation of the *c-fos *gene promoter, followed by Cre-recombination.

“Learning”-group mice showed a large number of eGFP-positive neurons in all the structures specifically related to FC learning [21], namely in the olfactory nuclei, hippocampus, amygdala, ventral thalamus, and in various neocortical areas: frontal association, prelimbic, infralimbic, cingulate, retrosplenial, parietal association, temporal association, entorhinal, somatosensory, auditory, visual, motor, insular, and orbital cortices (*Fig. 2 B,D,F,H*). At the same time, almost no eGFP-positive cells were detected in the aforementioned brain regions in the mice of the control group (*Fig. 2 A,C,E,G*). Rare eGFP-positive cells were detected in the control animals only in some areas of the neocortex: frontal association, dorsolateral, orbital, motor, somatosensory, pyriform, and ectorhinal. These results indicate that the method of genetic labeling of active neurons in Fos-Cre-eGFP mice enables successful visualization of the activation of a widely distributed population of neurons, involving a large number of various brain structures.

**Determining the phenotype of cells undergoing experience-dependent Cre-recombination**

In the experiment aimed at determining the type of cells undergoing experience-induced Cre-recombination, mice were injected with tamoxifen and then placed into the new context A. In 3 days, pairs of markers (eGFP and one of the specific cell type markers) were identified.

**Fig. 3:**
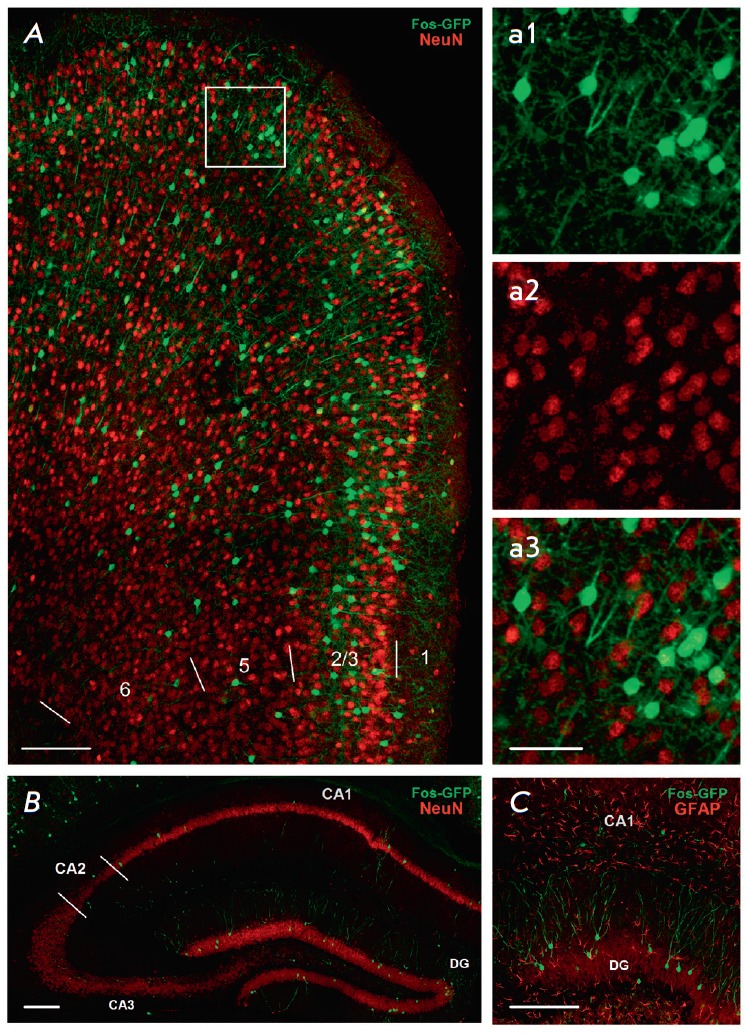
Experience-dependent Cre-recombination takes place only in neurons. Green channel – eGFP. Red channel – NeuN neuronal marker (A, B) or astrocyte marker GFAP (C). A – neocortex (frontal association cortex); B, C – dorsal hippocampus; scale: 150 μm. Microphotographs a1, a2, a3 show the area framed in A, separately in channels: a1 – cytoplasmic eGFP staining, a2 – nuclear NeuN staining, a3 – combination of the two; scale: 70 μm.

The NeuN protein, which is localized in the nuclei of virtually all types of neurons in the central nervous system of mammals, except for the Purkinje cells of the cerebellum, mitral cells of the olfactory bulb, and photoreceptor retinal cells, was selected as the marker of mature neurons [22]. The GFAP protein was selected as a cellular marker of astrocytes [23]. In all the examined structures, all eGFP-positive cells were also NeuN-positive (*Fig. 3 A,B*); there were no cases of eGFP colocalization with the GFAP protein (*Fig. 3B*).

Brain neurons can be classified as excitatory (pyramidal) and inhibitory (interneurons). CaMKII may be used as a marker of excitatory neurons. This enzyme is involved in the functions of excitatory synapses and is never synthesized in GABAergic interneurons [24]. Interneurons are highly variable in their morphological, electrophysiological, and biochemical properties, and, therefore, they are classified into several different types, for example, based on the synthesis of specific biochemical markers: PV, SOM, and NPY [25]. We evaluated colocalization of the eGFP protein and the marker of excitatory pyramidal neurons CaMKII or one of the markers of inhibitory interneurons (PV, SOM, and NPY) to determine which neuronal type corresponds to the cells that undergo Cre-recombination.

**Fig. 4:**
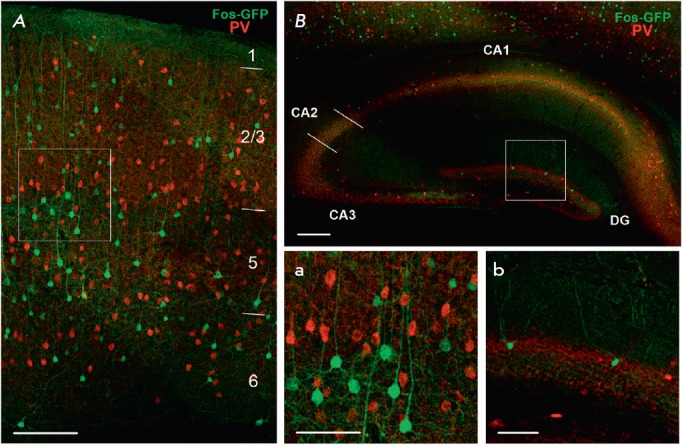
Combined staining for eGFP (neurons that underwent experience-dependent Cre-recombination) and interneuron marker parvalbumin (PV, red channel). A – neocortex (frontal association cortex), neocortical layers are numbered; B – dorsal hippocampus; scale: 150 μm. Microphotographs a and b show the neocortical and hippocampal areas framed in A and B, respectively; scale: 70 μm. CA1 – CA1 area of the hippocampus; CA2 – CA2 area of the hippocampus; CA3 – CA3 area of the hippocampus; DG – dentate gyrus of the hippocampus.

**Fig. 5:**
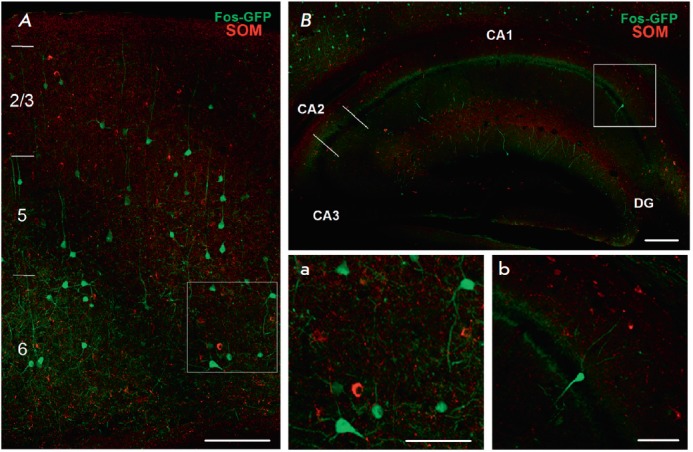
Combined staining for eGFP (neurons that underwent experience-dependent Cre-recombination) and interneuron marker somatostatin (SOM, red channel). A – neocortex (frontal associative cortex), neocortical layers are numbered; B – dorsal hippocampus; scale: 150 μm. Microphotographs a and b depict the neocortical and hippocampal areas framed in A and B, respectively; scale: 70 μm. CA1 – CA1 area of the hippocampus; CA2 – CA2 area of the hippocampus; CA3 – CA3 area of the hippocampus; DG – dentate gyrus of the hippocampus.

**Fig. 6:**
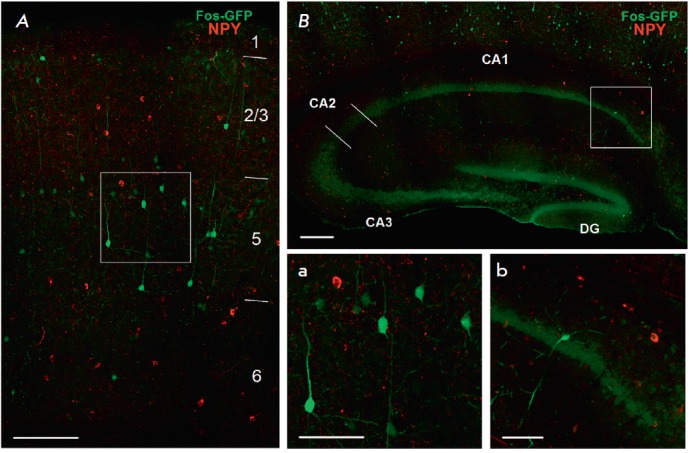
Combined staining for eGFP (neurons that underwent experience-dependent Cre-recombination) and interneuron marker neuropeptide Y (NPY, red channel). A – neocortex (frontal association cortex), neocortical layers are numbered; B – dorsal hippocampus; scale: 150 μm. Microphotographs a and b depict the neocortical and hippocampal areas framed in A and B, respectively; scale: 70 μm. CA1 – CA1 area of the hippocampus; CA2 – CA2 area of the hippocampus; CA3 – CA3 area of the hippocampus; DG – dentate gyrus of the hippocampus.

**Fig. 7:**
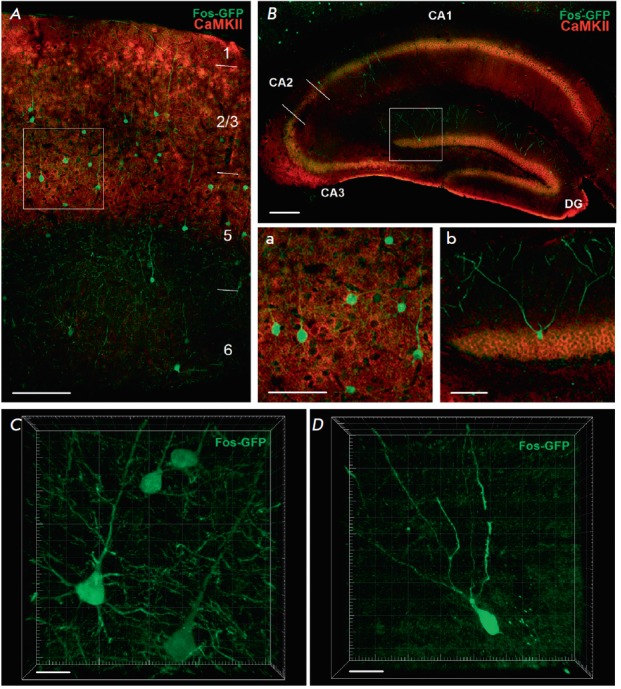
Colocalization of the eGFP-positive neurons that underwent experience-dependent Cre-recombination (green channel) with the pyramidal neuronal marker CaMKII (red channel). A – neocortex (frontal association cortex), numbers indicate neocortical layers; B – dorsal hippocampus, areas CA1, CA2, CA3 and dentate gyrus (DG); scale: 150 μm. Microphotographs a and b show the neocortical and hippocampal areas framed in A and B, respectively; scale: 70 μm. C and D – microphotographs of several neurons that underwent experience-dependent Cre-recombination in the neocortex and the hippocampus, respectively. Dendritic trees and axons branching out from soma are visible; scale: 20 μm. Colocalization of the eGFP-positive neurons that underwent experience-dependent Cre-recombination (green channel) with the pyramidal neuronal marker CaMKII (red channel). A – neocortex (frontal association cortex), numbers indicate neocortical layers; B – dorsal hippocampus, areas CA1, CA2, CA3 and dentate gyrus (DG); scale: 150 μm. Microphotographs a and b show the neocortical and hippocampal areas framed in A and B, respectively; scale: 70 μm. C and D – microphotographs of several neurons that underwent experience-dependent Cre-recombination in the neocortex and the hippocampus, respectively. Dendritic trees and axons branching out from soma are visible; scale: 20 μm.

We found no colocalization of eGFP with any of the interneuron markers (*Fig. 4*, *Fig. 5*, *Fig. 6*). At the same time, eGFP-positive neurons were also CaMKII-positive; i.e., they belonged to the class of excitatory pyramidal cells (*Fig. 7*). CaMKII-positive eGFP-positive neurons were detected in various layers of the neocortex (*Fig. 7A*), as well as in the pyramidal layer of the CA1 zone and the granular layer of the hippocampal dentate gyrus (*Fig. 7B*). Immunohistochemical detection of the eGFP protein showed staining of not only cell bodies, but also a significant part of the processes. Visual analysis of the morphology of these eGFP-positive cells also confirmed the conclusion that experience-induced Cre-recombination occurred in pyramidal cells (*Fig. 7 C,D*).

Therefore, molecular phenotyping of the cells showed that the experience-induced Cre-recombination occurs only in neurons, but not in the glial cells of Fos-Cre-eGFP mice, and that the neurons undergoing Cre-recombination belong to the family of excitatory pyramidal cells.

**Determining the proportion of cells that undergo Cre-recombination in animals after they explore a new environment**

The number of eGFP-positive neurons was assessed in different layers of the frontal association cortex in mice that had explored the context after tamoxifen injection (*n *= 5) in order to determine the proportion of neurons undergoing experience-induced Cre-recombination when the animals gained new experience and to analyze the patterns of distribution of those cells in the brain. Concomitant detection of the eGFP and NeuN mature neuron marker was carried out to assess the percentage of eGFP-positive cells among all the neurons in this layer [22].

**Fig. 8:**
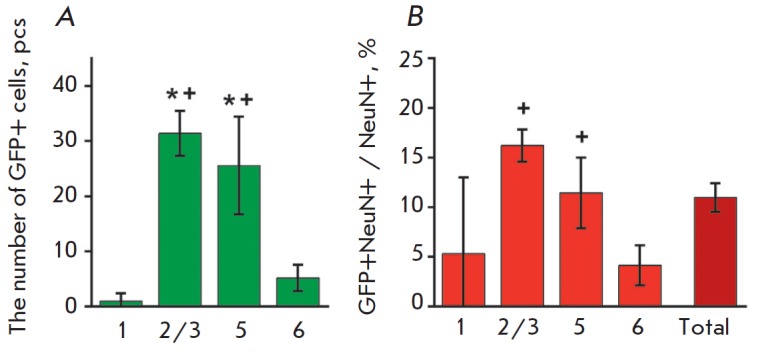
Analysis of the distribution patterns of the neurons that underwent experience-dependent Cre-recombination after exploration of a new context by layers of the frontal association cortex. A – the number of eGFP-positive cells; B – the proportion of eGFP-positive cells among all NeuN-labeled neurons. The numbers indicate neocortical layers, “total” – the value averaged over the whole frontal associative cortex. * – p < 0.02, + – p < 0.02 compared to layers 1 and 6, respectively, Sidak t-test. Data are shown as a mean value ± 95% CI

eGFP-positive cells were found in all the layers of the frontal association cortex, but their number was highest in layers 2/3 and 5 (F(1.298,5.191) = 41.47, *p *= 0.0009; pairwise comparison of layers 2/3 and 5 with other layers: *p* < 0.02), whereas only rare neurons that underwent Cre-recombination were detected in layer 1 (*Fig. 8A*). At the same time, the proportion of eGFP-positive cells among all the NeuN-labeled neurons was also highest in layers 2/3 and 5 (F(1.203,4.812) = 7.122, *p *= 0.0425; pairwise comparisons of layers 2/3 and 5 with layer 6: *p* < 0.02), and it averaged 11.0% for the entire frontal association cortex (*Fig. 8B*).

**The use of Cre-recombination to identify two populations of cognitively active neurons in one brain**

Cre-recombinase activity leads to permanent labeling of the neurons that are active within the time window defined by the action of tamoxifen. This opens the possibility of repeated placement of an animal with genetically labeled neurons in the situation of acquiring a new experience or reactivation of a previous one, followed by the detection of two separate populations of neurons activated upon two different cognitive loads within one brain. In this case, the neurons that underwent Cre-recombination after the first cognitive episode can be detected based on the presence of the eGFP protein and the neurons activated after the second episode can be visualized using immunohistochemistry based on the presence of the c-Fos protein.

**Fig. 9:**
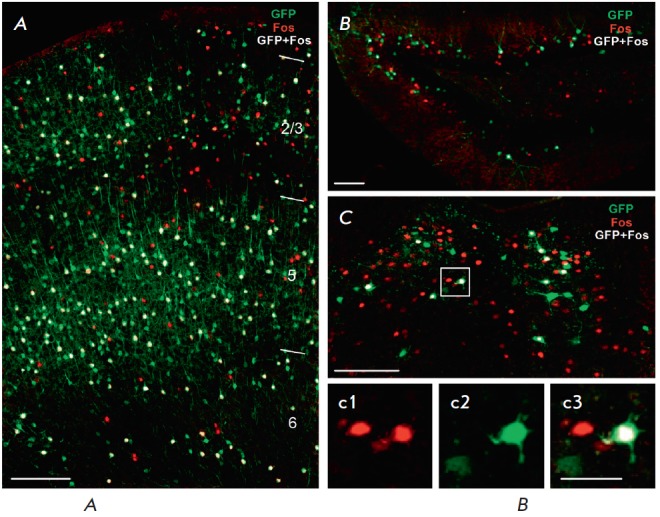
Immunohistochemical identification of the neurons activated during two cognitive episodes. Neurons activated during the first episode, exploration of a new environment, are labeled with eGFP (green channel); cells activated during the second episode, electric shock, are labeled with endogenous c-Fos protein (red channel). White pseudocolor labels the cells that are both eGFP- and c-Fos-positive, meaning that they were active in both cognitive episodes. A – neocortex (frontal association cortex), numbers indicate neocortical layers; B – dentate gyrus of the hippocampus; C – paraventricular thalamic nucleus; scale: 100 μm. Microphotographs c1, c2, c3 show the area framed in C, separately in channels: a1 – cytoplasmic eGFP staining, a2 – nuclear c-Fos staining, a3 – combination of the two; scale: 70 μm.

A high degree of overlap of neuronal populations labeled in mice that explored a new context (the first cognitive episode) and those that underwent subsequent immediate FS in the same context 25 min later (the second cognitive episode) was previously detected in the frontal association cortex. In our experiment, the mice were allowed to explore a new context after administration of tamoxifen and, 3 days later, received an immediate FS in order to visualize the two populations of neurons involved in the various cognitive episodes. Double immunohistochemical staining was used to detect the neurons activated when exploring the context (using eGFP protein) or receiving immediate FS (using c-Fos protein), as well as the neurons that were active in both cognitive episodes (eGFP and c-Fos) in various regions of the mouse brain (*Fig. 9*).

**Fig. 10:**
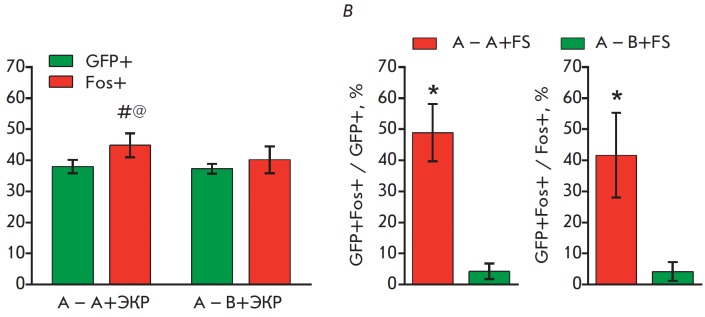
Quantitative analysis of the overlap between neuronal populations of the frontal association cortex that were active during two cognitive episodes. A – neuronal populations active during exploration of context A (eGFP-positive cells) and immediate shock (c-Fos-positive cells); B – proportion of neurons active during both cognitive episodes among all the neurons involved in the new context exploration (GFP+Fos+ / GFP+) or all the neurons involved in immediate shock (GFP+Fos+ / Fos+). # – p = 0.0192 compared to c-Fos-positive cells for “A – A+FS”, @ – p = 0.0212 compared to “B – A+FS”, Sidak t-test; * – p < 0.0001 compared to “B – A+FS”, Student’s t-test. Data are shown as mean values ± 95% CI

We assessed the number of neurons that were activated when exploring the context with application of subsequent immediate FS, as well as the overlap of these neuronal populations in the frontal associative cortex of A – A + FS group mice and control A – B + FS group mice (*Fig. 10*). Exploration of the new context and application of immediate FS activated similar-in-size populations of neurons in the frontal association cortex. However, a larger number of neurons were activated when FS was applied in the previously explored context A than in the new context B (“group” factor: F(1, 8) = 12.33, *p *= 0.0080; “cognitive episode” factor: F (1,8) = 11.37, *p *= 0.0098; interaction between factors: F (1,8) = 3.947, *p *= 0.0404; comparison of the number of c-Fos-positive cells in the groups A – A + FC and A – B + FC: *p *= 0.0212), *Fig. 10A*. In addition, FS application in a familiar context activated more neurons than it did in its initial exploration (comparing the number of c-Fos-positive and eGFP-positive cells in the group A – A + FS: *p *= 0.01924). In this case, the proportion of neurons activated when exploring the new context and then re-activated when applying FS was significantly higher in the group A – A + FS (48.8%) than in the group A – B + FS (4.2%), *p * < 0.0001. A similar result was obtained for the proportion of neurons activated in both cognitive episodes among all neurons activated upon application of FS (*Fig. 10B*).

The results of this experiment indicate that it is possible to use Cre-induced genetic labelling of neurons to identify and then analyze the populations of neurons activated in the same brain during two different cognitive episodes.

Previously, a transgenic technology based on the tTA-tetO system was used to label two populations of cognitively active neurons. Rejmers et al. used the tTA-tetO system to analyze overlapping of neuronal populations activated during memory formation and reactivation [27]. Two different genetic markers of transcriptional cell activation, *c-fos *and *zif268, *were used to compare these populations. However, it is known that these two genes have different baseline expression levels, different cellular functions, different specificities with respect to brain cell types, and that they are activated in response to different types of cognitive activity by animals [28]. Therefore, the comparison of the involvement of neuronal populations in two cognitive activity episodes for these two distinct markers poses great theoretical difficulties and draws serious objections. Furthermore, the tTA-tetO system has a number of methodological limitations: tTA-tetO transgenic mice require lifetime administration of doxycycline, while genetic labeling of neurons is possible only during the period of drug withdrawal. At the same time, the system activation time window after doxycycline withdrawal can take several days, which leads to a large number of nonspecifically marked neurons [27, 29]. Therefore, the transgenic system with Cre-recombinase seems to be more adequate for application in experiments aimed at identifying two neuronal networks activated in the same brain in different cognitive episodes.

## CONCLUSION

We generated bitransgenic Fos-Cre-eGFP mice in which experience-induced Cre-recombination resulted in genetic labelling of the neurons active during the action of tamoxifen. These mice demonstrated a low baseline level of Cre-recombination in a quiet state and significant increase in the number of eGFP-expressing genetically labelled neurons after acquisition of a new experience. In these mice, experience-induced Cre-recombination occurred in a large number of brain structures. Cre-recombination occurred in pyramidal excitatory neurons, but not in inhibitory interneurons. We also showed that Cre-induced genetic labelling of neuronal networks can be successfully used to identify activity by two different neuronal populations associated with different cognitive episodes within one nervous system and also to analyze overlapping of these populations of neurons.

## Abbreviations

eGFP: enhanced green fluorescent protein
GFAP: glial fibrillary acidic protein
NeuN: neuronal nuclei
CaMKII: Ca2+/calmodulin-dependent protein kinase II
SOM: somatostatin
NPY: neuropeptide Y
PV: parvalbumin
IEGs: immediate early genes
PCR: polymerase chain reaction
FC: fear conditioning
FS: footshock
